# Gross and Histopathology of Goats Feeding on *Opuntia stricta* in Laikipia County, Kenya

**DOI:** 10.1155/2021/8831996

**Published:** 2021-02-03

**Authors:** Jackson M. Ncebere, Paul G. Mbuthia, Robert M. Waruiru, Peter K. Gathumbi

**Affiliations:** ^1^University of Nairobi, College of Agriculture and Veterinary Sciences, Department of Veterinary Pathology, Microbiology and Parasitology, P.O. Box 29053-00625, Kangemi, Nairobi, Kenya; ^2^Directorate of Veterinary Services, Laikipia County, P.O. Box 31-10400, Nanyuki, Kenya

## Abstract

Various plant species such as *Opuntia stricta* have developed defensive measures, namely, spines, thorns, and other sharp pointed structures to protect themselves from herbivores and other animals feeding on them. *Opuntia stricta* has invaded the northern part of Laikipia County, Kenya, and its fruits are protected by small spines called glochids. This study determined the pathology in goats feeding on this plant in Laikipia County. Eighteen goats that had eaten the plant and six others that were raised in a ranch without *O. stricta* were purchased for the study. All study animals were clinically examined for lesions and euthanized for necropsy examination. Clinically, goats affected by *O. stricta* had poor body condition, wounds on various body parts, and diarrhea. Variable numbers of *O. stricta* spines occurred externally on the skin throughout the body and elicited pain, swelling, and ulcerative wounds on affected parts. Internal lesions were observed in subcutaneous tissues (100%), together with stomatitis, cheilitis, gingivitis, glossitis, abomasitis (100%), rumen, reticulum, omasum thinning and loss of papillae (72.2%), esophagitis, and duodenitis (5.6%). Carcasses had gelatinous fat and muscular atrophy. Other gross lesions were generalized viscera atrophy, edema, subcutaneous emphysema, lymphadenopathy, abscesses, ascites, hydrothorax, and hydropericardium. The abomasum wall and its mucosal folds were swollen with edema, haemorrhages, and scattered foci of abscesses. Histopathology confirmed the main lesions in all affected goats were foreign-body granulomas which were located in all organs with gross lesions. Goats from *O. stricta*-free ranches had no spines or lesions. The pathological effects caused by *O. stricta* resulted in emaciated goats due to pain, inability to masticate and assimilate food, and stress, resulting in poor carcass and organs quality and possible condemnation and death. This could affect the socioeconomics and livelihoods of communities in the study area, and therefore, the spread of this plant needs to be controlled.

## 1. Introduction

**Figure 1:**
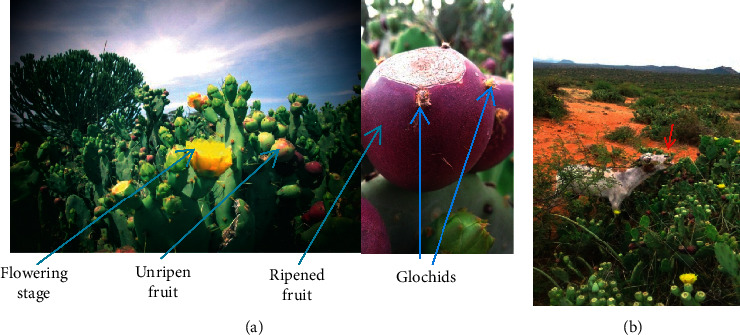
*Opuntia stricta* in Laikipia County rangelands (a) showing fruits at various stages of development and hairy thorns (glochids) on the fruits. A goat browsing (red arrow) on fruits of *Opuntia stricta* in Laikipia North sub-County (b).

*Opuntia stricta* variety *stricta* (Haw.) in the family *Cactaceae* and genus *Opuntia* is one of the plants with thorns and spines [1], and it has invaded the northern part of Laikipia County, Kenya. It has reduced prime grazing land and access to grass [2, 3]. Its cladodes and fruits are protected by thorns and small spines called glochids. Goats, other livestock, and wild animals feed on the ripened fruits, especially during the dry seasons (Figure 1).

This results in attachment of glochids on the skin surfaces and gastrointestinal tract of the animals. These spines injure and cause wounds on points of their attachments. The affected animals become emaciated, have eye problems, show reduced milk production, and eventually die [2]. The cause of progressive loss of body condition and subsequent death of goats feeding on spine-laden *O. stricta* fruits has not been determined, making it difficult to institute meaningful intervention to manage animals that suffer from *O. stricta*-related conditions. The aim of this study, therefore, was to determine and document pathological lesions and their distribution in body organs of goats feeding on *O. stricta* in Laikipia County, Kenya, in order to explain its effects on the health of livestock that feed on it.

## 2. Materials and Methods

### 2.1. Study Site

**Figure 2:**
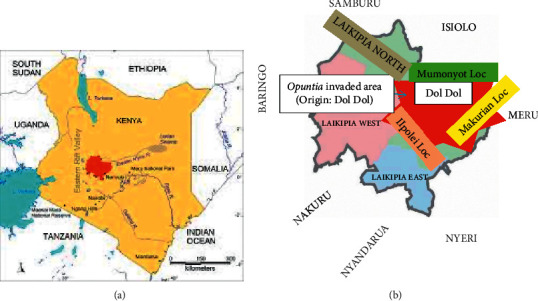
Map of Kenya showing the location of Laikipia County (a). Map of Laikipia County showing three locations in Laikipia North sub-County invaded by *Opuntia stricta* (b).

The study was carried out in Laikipia North sub-County, Laikipia County in Kenya, between October 2018 and February 2019. Laikipia North sub-County is a purely pastoral zone, and part of it has been invaded by *O. stricta* plants (Figures 2(a) and 2(b)).

### 2.2. Study Goats

**Figure 3:**
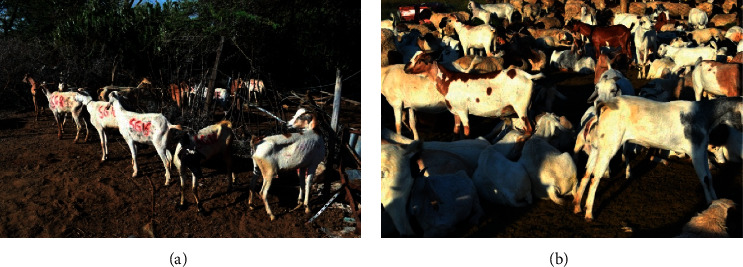
(a) Goats in poor body condition (sick goats) that had consumed *Opuntia stricta* in Laikipia North sub-County. (b) Goats in good body condition (controls) that had not consumed *Opuntia stricta* in a ranch in Laikipia North sub-County.

The use of animals in this study was approved by the Biosafety, Animal Use, and Ethics Committee of the Faculty of Veterinary Medicine, University of Nairobi, Ref: FVMBAUEC/2019/192. Random purposive sampling was used in the selection of adult study goats [4, 5]. Twenty-four (24) small East African goats were purchased for the study. Among these goats, 18 were raised in pasture with abundant growth of the plant and had eaten *O. stricta* (sick goats) (Figure 3(a)) and 6 were raised in pastures that did not have the plant (Figure 3(b)) and had not eaten the plant (controls).

The sick goats were identified from the rest of the animals in the herd as having been affected by *O. stricta* based on their manifestation of unique clinical signs associated with the condition, as recognised by pastoralists in the study area. These signs were poor body condition, presence of *O. stricta* spines on the skin and occurrence of red colouration on the lips and hair coat due to ripened *O. stricta* fruits, eye problems, and wounds on the lips and oral mucosa. The 18 sick goats were purchased from Mumonyont, Makurian, and Ilpolei locations that are invaded by *O. stricta* in Laikipia North sub-County. The *O. stricta*-affected goats were purchased in batches of six goats per location, three goats per household (“manyattas”) that were located about 15 kilometers apart within one location. The households were randomly selected to ensure that *O. stricta*-affected goats were distributed across the study area. All sick goats were gathered in one of the households in nearby Dol Dol town in the Mukogodo location and provided adequate care and welfare before they were humanely killed at the Dol Dol slaughterhouse. The 6 goats that had not eaten the plant (controls) were purchased from two *O. stricta*-free ranches (three per ranch) from the Mpala location in the same locality.

All control goats were kept within one of the ranches and then transported for humane killing at the Nanyuki slaughterhouse, which was the most proximate to the ranches. Each animal was identified with a number (SG1 to SG24).

### 2.3. Antemortem Physical Examination of the Study Goats

Thorough physical examination was carried out to determine the external lesions that may have been caused by *O. stricta* on each goat. The percentage occurrence, type, and location of each external lesion were determined. The body condition score was determined on a scale of 1 to 5 for each goat, where one represented very emaciated goat and 5, goat in a very good body condition. The body condition score was determined using the method described by Villaquiran et al. [6].

### 2.4. Euthanasia of the Study Goats

A penetrating captive bolt gun loaded with cartridges sized 0.22 mm (blank pink cartridges) was used to stun the study goats. The optimal anatomic site and direction of a penetrating captive bolt was determined as described by Plummer et al. [7]. Immediately after stunning, a deep transverse cut across the throat behind the jaws was made using a sharp knife to sever the two carotid arteries and the two jugular veins effectively. Goats were euthanized, one at a time for a detailed postmortem examination [8, 9]. Four goats were humanely killed per day to enable thorough examination of carcasses.

### 2.5. Necropsy Examination

Skinning was carefully performed observing all lesions on the external surface of the skin and subcutaneous tissue. Gross lesions and their distribution were described for each body organ system. A self-evaluation criterion (Table 1) was used to score severity of lesions as mild, acute, subacute, and chronic in goats which had consumed *O. stricta*.

After postmortem examination, carcasses were disposed into the condemnation pits located within the slaughterhouse compound. Tissues for histopathology were collected from the earlobes, lips, tongue, esophagus, rumen, reticulum, abomasum, duodenum, small and large intestines, and lymph nodes and fixed in 10% neutral buffered formalin solution.

### 2.6. Histopathology

Formalin-fixed tissues from twelve (12) out of twenty-four (24) study goats were processed for histopathology using standard procedures and stained with haematoxylin and eosin (H&E) as described by Slaoui and Fiette [10]. Nine out of the twelve goats were those that had consumed *O. stricta*. These were selected on the basis of the severity of external and internal gross lesions (chronic, subacute, or mild). The remaining three goats were from the control group. Histopathology slides were examined using an Olympus microscope mounted with a live-view digital SLR camera (E-330), and photomicrographs were taken.

### 2.7. Statistical Analysis

Data on external and internal lesions were entered into Microsoft Excel sheet, cleaned, verified, and validated. The data were then imported into Statistical Package for Social Sciences (SPSS) version 22 for analysis. Descriptive statistics were run to get the percentages of lesions in each organ or body systems of goats affected by the consumption of *O. stricta.* Mean body condition scores of all goats were determined. An independent samples t-test was carried out to determine differences in the mean body condition scores of the study animals.

## 3. Results

### 3.1. Body Condition Score

Goats that had consumed *O. stricta* had a mean body condition score of 1.028 ± 0.1179 while those which had not consumed the plant (controls) had a mean body condition score of 4.417 ± 0.2041. The independent samples *t*-test showed that there was a significant difference between the body condition scores of the two study groups (*t* = -50.579, df = 22 and *p* < 0.001).

### 3.2. External Gross Lesions

Table 2 summarizes externally located gross lesions observed, their distributions, and frequency of occurrence (percentages) in goats that were reared in *O. stricta*-invaded locations. Goats that had fed on *O. stricta* were in pain as they walked, emaciated, dull, had hunched backs, lame, rough hair coat, and soiled perineal region due to diarrhea.

**Figure 4:**
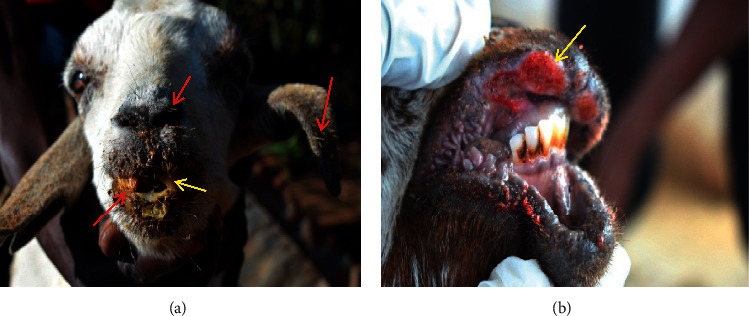
Septic ulcerative wounds (yellow arrows) on the lips, gum, and oral mucosa (a and b); nasal and oral mucopurulent discharges in a goat unable to close its oral cavity due to severe wounds and many spines of *Opuntia stricta* on the oral mucosa (pear mouth-like condition) and spines in the ear lobes and nostrils (a) (red arrows).

Spines of *O. stricta* had attached on the entire skin, over the ear lobes (making them swollen and ulcerative), eyes, nostrils, and lips of all goats, (100%) causing dermatitis, severe otitis externa, cataract, blepharitis, keratoconjunctivitis, rhinitis, and cheilitis (Figure 4). A majority of the affected goats (94.4%) had ulcerative wounds on the lips (Figure 4). Some of the affected goats (33.3%) had oral lesions (stomatitis, gingivitis, and glossitis) characterized by swellings, lacerations, wounds, and ulcers and could not close the oral cavity or chew cuds (Figure 4(a)).

**Figure 5:**
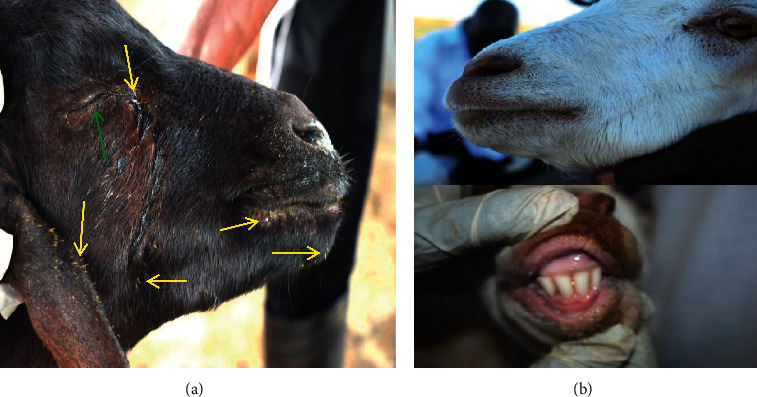
Spines on the ear lobe, face, eye lid, and lips (yellow arrows) and closed blind eye with ocular discharge (green arrow) (a) of goats that had consumed *Opuntia stricta*. Goats from *Opuntia stricta*-free ranches with clean face, eyes, lips, gum, and oral cavity with no observable spines on the external body parts (b).

Eye lesions (keratitis, conjunctivitis, and cataract) occurred in 100% of the goats. Some had cataract in one eye with concurrent blindness (16.7%) or had cataracts in both the eyes (22.2%) and were blind while others had flowing mucopurulent eye discharges (Figure 5(a)).

Some goats had mucopurulent oral and nasal discharges. The presence of red colour of ripened *O. stricta* fruit juice occurred on the lips and hair coat in 44.4% of animals, an evidence that goats had consumed or were in contact with the plant. Other external lesions observed were swollen lymph nodes, namely, prescapular (27.8%), precrural (11.1%), and parotid (27.8%). Subcutaneous abscesses of varying sizes occurred in different locations on the skin of affected goats (22.2%). The subcutaneous abscesses were swollen, painful, and tender. They measured 1 cm to 3 cm in diameter.

Goats from *O. stricta*-free ranches were in good body condition with well-groomed hair coat, clean eyes, face, lips, and oral mucosa and had no observable spines or associated lesions on external body parts.

### 3.3. Internal Gross Lesions

**Figure 6:**
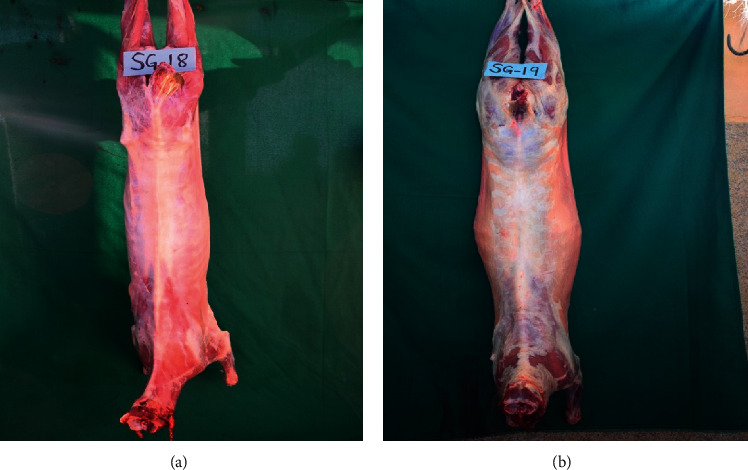
(a) Emaciated carcass showing atrophied musculature and body fat and the prominent vertebrae column of a goat that had consumed *Opuntia stricta* and (b) a fat carcass of a goat that had not consumed *Opuntia stricta*.

Carcasses of all goats which had consumed *O. stricta* were emaciated and lean, and there was subcutaneous emphysema. Their body fat was atrophied and gelatinized; musculature had also atrophied (Figure 6(a)). Carcasses of the goats which had not consumed the plant were fleshy and well covered with fat (Figure 6(b)).

These lesions in affected goats are summarized in Table 3. These goats had also generalized visceral atrophy, variable amounts of ascites, hydrothorax, and hydropericardium. Thorns and hairy spines were attached and caused glossitis (especially on the median sulcus), gingivitis, and stomatitis in all goats (100%).

**Figure 7:**
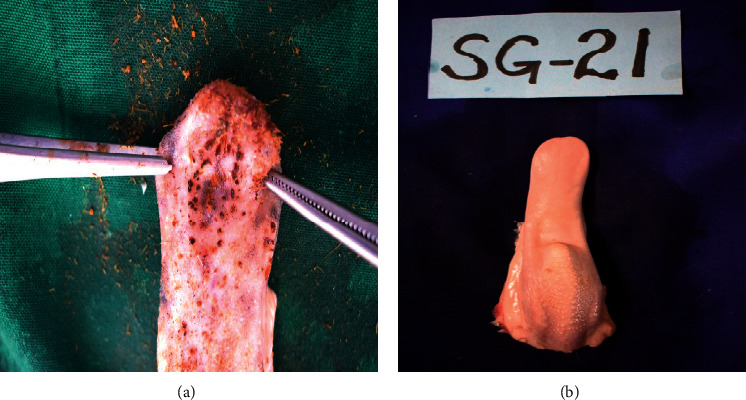
(a) Glossitis characterized by ulceration and swollen tongue of a goat that had consumed *Opuntia stricta* and (b) a healthy-appearing tongue of a goat that had not consumed *Opuntia stricta*.

Ulcerative cheilitis, gingivitis, and stomatitis occurred in 94.4% and glossitis occurred in 83.3% (Figure 7(a)) in goats that had consumed the plant, compared with those that had not eaten (Figure 7(b)). Some affected goats (44.4%) had missing (oligodontia) or worn out teeth.

Spines of *O. stricta* occurred in the esophagus (5.6%) and 72.2% in the rumen-reticulum-omasum and 5.6% in the duodenum of these goats. Thorns and spines in the forestomachs did not cause overt gross pathology except reduction in numbers and size of the rumen papillae in 16.7% of affected goats.

**Figure 8:**
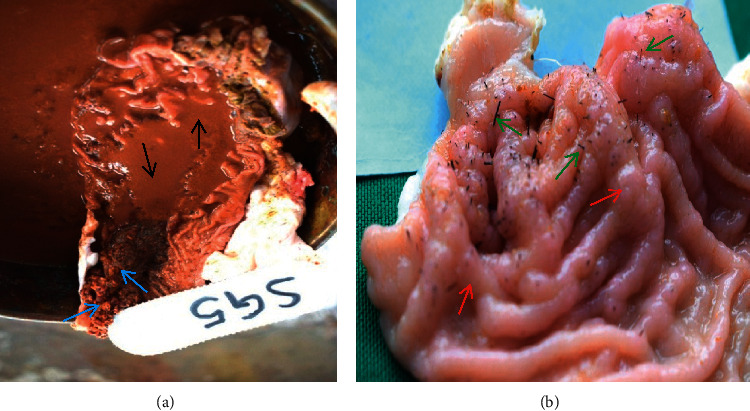
(a) Abomasitis showing dark-tan-coloured abomasal contents at the fundus and corpus regions of abomasum (black arrows) and seeds of *Opuntia stricta* fruits packed at the pyloric region (blue arrows). (b) Another case of abomasitis showing thorns and spines embedded into the mucosa (green arrows) and thickened abomasal folds (red arrows) of goats that had consumed *Opuntia stricta*.

All affected goats had thorns, spines, and lesions in the abomasum (100%) causing variable forms of abomasitis. Abomasal folds and glands were swollen as a result of the reaction to injuries inflicted by thorns and spines (Figure 8(b)). The abomasa were filled with fluid contents which were dark tan in colour at the fundus and corpus regions (Figure 8(a)). The pyloric sphincter restricted passage of *O. stricta* seeds, thorns, and spines. These were consequently firmly packed in the pylorus region (Figure 8(a)).

**Figure 9:**
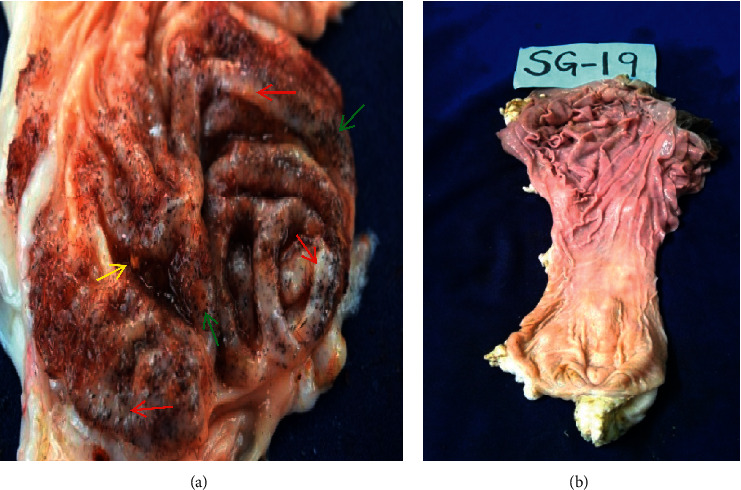
(a) Abomasitis showing severe inflammation characterized by swollen folds with edema, abscesses (red arrows), *Opuntia stricta* seeds lodged between folds (yellow arrow), and numerous thorns and spines (green arrows) of a goat that had consumed *Opuntia stricta*. (b) Abomasum of a goat that had not consumed *Opuntia stricta* showing clean fundus, corpus, and pyloric areas, free of thorns and spines.

Thorns and spines that penetrated into the abomasal mucosa caused irritation and severe abomasitis that was characterized by edema and swollen folds; some had haemorrhages and multifocal soft abscesses measuring 2 mm to 5 mm in the longest diameter on the wall Figure 9(a).

*Opuntia stricta*-associated lesions were of variable severity on the lips, oral mucosa, and abomasum of affected goats. These lesions were mild, moderate or severe, and subacute or chronic. Severe lesions on the oral mucosa, lips, and abomasum were seen in 72.2% of affected goats. All goats with severe stomatitis had correspondingly severe abomasitis. The rest (27.8%) had mild stomatitis and abomasitis. There were no moderate lesions. Subacute cases had massive spines penetrating the abomasal mucosa resulting in abomasitis characterized by marked haemorrhages, edematous and thick mucosal folds, hypertrophied glands, and the presence of dark-tan fluid in the lumen of the abomasum. Mild cases had almost normal abomasal mucosa that had few thorns and spines, and their mucosa was stained reddish by recently swallowed ripened *O. stricta* fruit juice. Chronic cases had uncountable spines embedded into the abomasal mucosa resulting in abomasitis characterized by leathery appearance, hypertrophied glands, thick folds, and abscesses measuring 2 mm to 5 mm.

Other less frequent gross lesions in *O. stricta*-affected goats were the presence of thorns and spines in the subcutaneous tissues and muscles occurred in 5.6% of the affected goats and abscesses in kidneys (11.1%), livers (27.8%), lungs (5.6%), thoracic paravertebral areas ventral to the spinal cord (5.6%), and abdominal cavity (5.6%). On the cut surface, all these abscesses produced thick yellowish pus. The duodenum had enteritis in one goat, while small and large intestines of goats which had consumed *O. stricta* were empty of solid contents. They had thin walls and brown to pinkish red-stained watery contents probably caused by haemorrhages from *O. stricta*-inflicted wounds in the gastrointestinal tract augmented by the red colour of the ripened *O. stricta* fruits.

Goats which had not consumed *O. stricta* had no internal lesions. The oral cavities, abomasa, and other organs had neither thorns and spines nor evidence of gross lesions (Figures 5(b) and 9(b)).

## 4. Histopathological Lesions

Histopathological lesions were consistently observed in organs with gross lesions in all *O. stricta*-affected goats. External lesions occurred on the skin including the ears, external lips' surface, and mucocutaneous junction. Internal lesions mainly affected the oral mucosa, tongue, and abomasa. Regardless of the tissue affected, the typical microscopic lesions were foreign-body granulomas and the presence of sections of embedded thorn or spine of *O. stricta*. The spines appeared yellow-brown and provoked mononuclear inflammation characterized by the marked infiltration by lymphocytes, plasma cells, macrophages, and fibrosis. The histopathological lesions in some of the severest affected body parts are described below.

### 4.1. Ears and Lips

**Figure 10:**
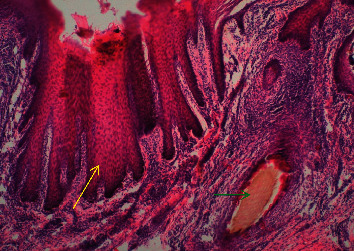
*Opuntia stricta* thorn (green arrow) deep in the ear muscles surrounded by mononuclear cells and granulation tissue. Elongated and expanded rete pegs (yellow arrow) and disrupted stratum corneum in severe chronic otitis externa (H&E x10).

Multiple foreign body granulomas occurred in the ears and lips. The affected ears and lips were characterized by marked thickening of all layers of the epidermis with elongated and expanded rete ridges (acanthosis) (Figure 10) and hyperkeratosis and parakeratosis of the stratum corneum.

**Figure 11:**
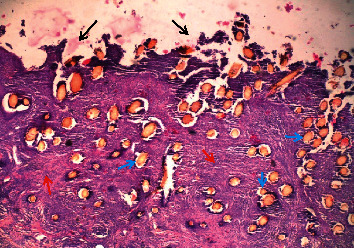
Numerous *Opuntia stricta* thorns deep in the muscles of the goat lip and the associated multifocal granulomas (blue arrows). The epidermal layer is necrotized and eroded (ulcerated) (black arrows), and skeletal muscles have been replaced by granulomatous tissue reaction (red arrows) in a goat with severe chronic cheilitis (H&E x40).

Some *O. stricta* thorns penetrated the ear to the level of cartilage. The soft tissues were destroyed and replaced by granulomatous reactions (Figure 11). There was increased mononuclear cell infiltration in the dermis in both ears and lips. Some thorns injured blood vessels in the areas causing haemorrhages. In both tissues, there was occasional disruption on the epithelia due to wounds and ulceration.

### 4.2. The Tongue

**Figure 12:**
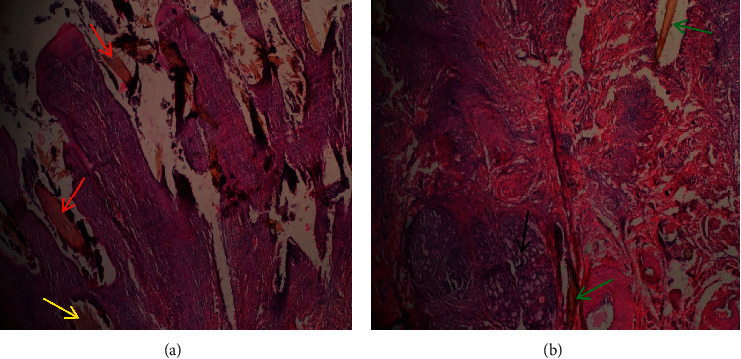
(a) Numerous *Opuntia stricta* thorns penetrating the tongue (red arrows), completely splitting, destroying, and disrupting the anatomical structure of the organ. Some thorns in the deep tongue muscles are surrounded by mononuclear cells in an acute severe glossitis (yellow arrow) (x10). (b) *Opuntia stricta* thorns deep in the tongue muscles (green arrows) affecting the sublingual glands in a goat (black arrow) with glossitis (x40).

Many *O. stricta* thorns were observed penetrating the tongue causing rupture and discontinuity of the mucosa, tongue muscles, and other tissues (Figure 12(a)). Some penetrated deep into the skeletal muscles, resulting in multiple coalescence of foreign-body granulomas. Scattered erosion, ulceration, and pressure atrophy of the mucosa and lingual papillae occurred. Some thorns penetrated deeper and formed granulomas adjacent to the sublingual glands (Figure 12(b)).

### 4.3. Abomasum

**Figure 13:**
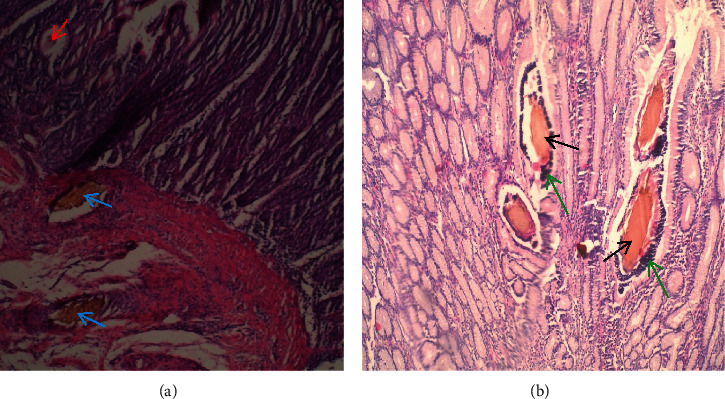
(a) Several *Opuntia stricta* thorns penetrating deep into the glandular mucosa (red arrow) and tunica muscularis (blue arrows) of the abomasum in mild abomasitis (x40). (b) *Opuntia stricta* thorns in the glandular mucosa of abomasum (black arrows) causing pressure atrophy of glands and attracting mononuclear cells infiltrations around them in mild case of abomasitis (green arrows) (x100).

Thorns were located mainly in tunica mucosa penetrating all layers, namely, lamina epithelialis, lamina propria, and stratum compactum and lamina muscularis mucosae. There was shredding of the mucosal epithelium into the abomasal lumen. Some thorns and spines extended to tunica muscularis (Figure 13(a)). Thorns and spines appeared as single, cylindrical, elongated, brownish-yellow bodies (cross section) or penetrating darts with a tapering end (longitudinal section). Foreign-body granulomatous reactions were observed surrounding each thorn or spine. In cases where the spines or thorns were many, the lesions were a mosaic of interconnected granulomas with a centrally located spinous body and marked bridging connective tissue. The highest load of spines or thorns was found on the glandular mucosa mostly near the lumen and deep in the mucosa affecting the lamina propria and gastric glands (Figure 13(b)).

**Figure 14:**
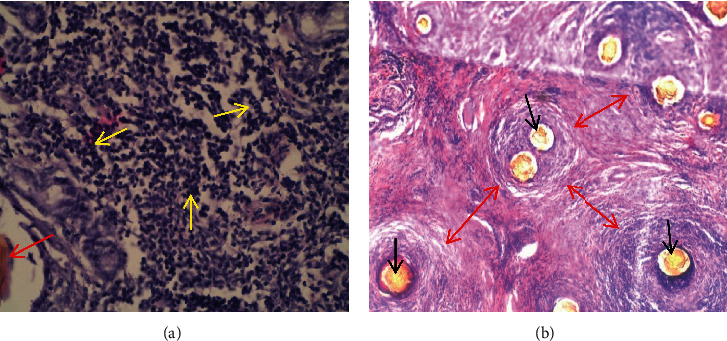
(a) Numerous mononuclear cells (yellow arrows), mainly macrophages, lymphocytes, and plasma cells, infiltrating areas where *Opuntia stricta* spines (red arrow) are located in severe abomasitis (x100). (b) Multifocal *O. stricta* thorns (Black arrows) and associated coalescing foreign-body granulomatous reactions (red arrows) in the pyloric region of a goat with severe chronic abomasitis (x40).

Granulomas caused pressure atrophy of the gastric glands. In the pylorus, multiple granulomas coalesced and the affected areas were fully covered by granulation tissue with multiple focal centers of thorns surrounded by mononuclear cells (Figures 14(a) and 14(b)). The spines and thorns had disrupted and mechanically destroyed the affected part of the tissue.

## 5. Discussion

*Opuntia stricta* variety *stricta*, plants with thorns and spines [1], is amongst 100 of the “World's Worst” invaders and toxic wild plant [11]. Shackleton et al. [2], through a questionnaire, assessed local pastoralists perception of *O. stricta* in Laikipia County. They reported that pastoralists felt that the plant contributed to ill health such as eye problems, swollen oral mucosa, poor body condition, and eventual death of livestock due to *O. stricta* glochids attachment. The pathology in affected livestock (especially goats) and its contribution to ill health were not well documented in that study.

In this study, the gross and histopathological changes and effects of this plant are described in goats that have browsed and fed on *O. stricta*. The type, distribution, and percentages of both external and internal lesions and carcass condition in goats after feeding on the *O. stricta* fruits and other parts with thorns and spines in their natural state [2] are hereby reported.

Goats in the study area feed on spiny *O. stricta* fruits in its natural state [2] and develop pathological lesions as observed in this study. This is different in other countries where this plant is fed to livestock after spines and thorns are removed using propane or paraffin flame treatment, and therefore, animals do not develop lesions [12]. No gross and microscopic lesions were observed in control goats that were reared in *O. stricta*-free ranches, indicating that spines and thorns were associated with lesions in affected goats in Laikipia North sub-County, Kenya.

Main pathological lesions were located externally on the head region (ears, face, eyes, and lips) and skin of the body and legs and internally in the oral cavity and entire gastrointestinal tracts in goats that had consumed *O. stricta*. Externally, the affected goats were emaciated, had hunched backs and rough hair coats, and lame, and some were blind while others were grinding teeth in pain. They had wounds on the ears, skin abscesses, eye cataract, blepharitis, keratoconjunctivitis with clear or mucopurulent discharges, cheilitis with or without ulcerations, clear to mucopurulent nasal and mouth discharges, and attached few to numerous thorns and spines on affected parts. The pain, movement limitations, and damage to affected tissues (blindness and cheilitis) could contribute to difficulties in goats finding feed, browsing, and stress, which can contribute to poor health of affected goats.

With their sharp tapering ends, the thorns and spines penetrated the skin into the deep body tissues such as subcutaneous tissue, lymph nodes, and skeletal muscles. Like other penetrating foreign bodies from the environment, they can introduce infectious (pyogenic) microorganisms as previously reported [13] such as *Corynebacterium pseudotuberculosis* [14–16]. Foreign body causing eye lesions are well known and are frequently encountered when thorns penetrate and inflict trauma to the ocular tissue [17]. Eye lesions and blindness that occurred in goats raised on *O. stricta*-invaded pastures were associated with thorn-inflicted traumatic injuries to the eye. Abscesses that occurred on the face, body flanks, legs, and in internal organs (lungs, liver, kidneys, thorax, along the ventral vertebral column, and abomasum) could have been due to effects of *O. stricta* spines as reported previously that sharp pointed plant structures can cause this [18–20]. Enlargement of draining lymph nodes may affect the animal's health and defense against infections and result in organs and carcass condemnation at slaughter as observed in the study.

Thorns inflicted severe damage to the oral cavity resulting in stomatitis, gingivitis, and glossitis resulting in severe irritation, painful ulcerations that prevented the animal from closing the oral cavity, ingestion, mastication, and chewing the cuds. This resulted in a pear mouth-like condition as reported in cattle in the USA [2] but not in goats. Some goats had lost or worn out teeth as reported for *Prosopis* spp. on other livestock due to thorn injuries [21]. Coexistence of severe lesions in the oral cavity further complicated the capacity of prehension and digestion functions and corroborates the loss in body condition and poor health that was observed in goats that were raised in *O. stricta*-invaded pastures.

Unlike in other reports where excessive consumption of cactus fruit is reported to results in constipation [22, 23], this was not present in the affected goats. However, there was reduction in number and size of papillae in fore stomachs in affected goats which could affect their functions.

*O. stricta* caused severe abomasitis, pain, thickening of the mucosa, and presence of multifocal abscesses which could compromise the functions of abomasum. This could imply that these spines might have been harboring some pyogenic microorganisms [12, 18–20, 24] or enabled entry of organisms from the gastrointestinal tract. In this study, seeds and thorns of *O. stricta* had impacted the pyloric area and sphincter restricting flow of ingesta; hence, only fluid contents were found in the intestines, resulting in diarrhoea. This has not been reported previously. Such effects could result in poor digestion and assimilation of food affecting remarkably the health of goats and contributed to overall loss in animal production in the affected rangelands.

Histopathology showed the presence of multiple thorns penetrating deep into the skin, earlobes, lips, tongue, and abomasum causing respective tissue damage followed by the formation of foreign-body granulomas which replaced destroyed body soft tissues. The foreign-body granulomatous lesions were composed of mononuclear cells, mainly macrophages and lymphocytes, and a few plasma cells as reported elsewhere in humans [25]. Granulomas are soft tissue responses normally provoked by insistent microorganisms or their products which resist phagocytosis or inactive foreign material [26]. They initiate the damage through a type IV hypersensitivity immune-mediated reaction [27]. Granulomas, therefore, differ in morphology and are triggered by diverse irritants [26]. Results of the present study agree with previous ones that *O. stricta*-associated foreign bodies in tissues cause noxious and sensitive responses including, infection and inflammation that vary in severity [25]. Lesions observed reduced the aesthetic value of meat and can cause condemnation of the carcasses and organs from goats which had consumed *O. stricta* at slaughter [28]. This could result in high economic losses to the pastoral community as reported in beef cattle in the USA with pear mouth and cactus tongue that rendered the condemnation of the latter [2]. In contrast, goats that had not consumed *O. stricta* (controls) had no lesions, and their carcasses were fat and full of flesh.

## 6. Conclusions

The study aimed to document pathology in goats consuming *O. stricta* in Laikipia North sub-County, Laikipia County, Kenya. Gross external and internal lesions and histopathological lesions in various organs and tissues, type, and prevalence have been documented. Consumed penetrating plants' thorns and spine were responsible for lesions observed on the skin, ears, oral cavity, and entire gastrointestinal tract. The extensive damage of these organs and tissues was responsible for the loss of both anatomical (emaciation, tissue, organ, and visceral atrophy) and physiological (prehension, mastication, assimilation, and digestion) functions and livestock deaths and losses in production. Measures to manage the spread of *O. stricta* in Laikipia County need to be instituted in order to prevent the associated impact on lives and livelihoods of communities living in the area.

## Figures and Tables

**Table 1:** Criteria used to score external and internal lesions of goats feeding on *Opuntia stricta* in Laikipia North sub-County.

Lesions	Classification
1 a) Dermatitis, otitis externa, eye problems, cheilitis, stomatitis, glossitis, and abomasitis due to 1–100 *O. stricta* thorns and spine attachments b) Small swellings at the *O. stricta* thorns and spine attachment sites without wounds on the skin, ears, eyes, lips, oral mucosa, tongue, and abomasum	Mild
2 a) Dermatitis, otitis externa, eye problems, cheilitis, stomatitis, glossitis, and abomasitis due to 100–200 *O. stricta* thorns and spine attachments b) Small reddened wounds measuring between 0.1 and 0.4 cm in diameter at the site of attachment by *O. stricta* on respective organ parts c) Abomasitis showing partially swollen glandular mucosa where thorns and spines are attached to abomasal folds	Acute
3 a) Dermatitis, otitis externa, eye problems, cheilitis, stomatitis, glossitis, and abomasitis due to numerous (over 200) *O. stricta* thorns and spine attachments b) Fresh wounds on the skins, lips, and oral mucosa measuring 0.5 cm or more in diameter c) Abomasitis with conspicuous thick mucosa folds and numerous swellings with haemorrhages where *O. stricta* thorns and spines are attached	Subacute
4 a) Dermatitis, otitis externa, eye problems, cheilitis, stomatitis, glossitis, and abomasitis due to uncountable *O. stricta* thorns and spines attachments b) Septic extensive ulcers measuring more than 0.5 cm in diameter on the skin, lips, and oral mucosa (some covering almost half of the affected lips and oral mucosa) c) Abomasitis with swollen glands and abscesses	Chronic

**Table 2:** External lesions observed on goats that had eaten *Opuntia stricta* in Laikipia North sub-County.

External lesion on goat that had eaten *Opuntia stricta*	Number of goats examined	Percent of goats with the lesion
Dermatitis (face, body trunk, and legs)	18	100.0
Otitis externa involving all ear lobes	18	100.0
Blepharitis	18	100.0
Cheilitis involving both the lips	18	100.0
Ulcerative cheilitis on the lips	17	94.4
Lymphadenopathy (parotid and prescapular lymph nodes)	5	27.8
Bilateral cataracts and blindness	4	22.2
Abscesses (face, body trunk, and legs)	4	22.2
Cataracts on one eye and blindness	3	16.7
Lymphadenopathy (precrural lymph nodes)	2	11.1

**Table 3:** Gross lesions in the internal organs of goats that had eaten *Opuntia stricta* in Laikipia North sub-County.

Type and location of gross lesions	Number of goats with the lesions	Percent of goats with lesions
Gingivitis and stomatitis	18	100.0
Abomasitis	18	100.0
Ulcerative stomatitis	17	94.4
Ulcerative glossitis	15	83.3
Reduced ruminal, reticular, and omasal papillae	13	72.2
Oligodontia and worn out teeth	8	44.4
Atrophied wall and reddish fluid in the small and large intestines	6	33.3
Liver abscesses	5	27.8
Kidneys abscesses	2	11.1
Duodenitis	1	5.6
Esophagitis	1	5.6
Abscesses in the lungs, abdominal cavity, thoracic cavity, and ventral side of vertebral column	1	5.6
Thorns and spines in the subcutaneous tissues and muscles causing myositis	1	5.6

## Data Availability

Data associated with this research article are available on request from the corresponding author.
